# Retatrutide Shows Multiple Metabolic Benefits in Diet‐Induced Obese MASH Mouse and Hamster Models

**DOI:** 10.1002/oby.70155

**Published:** 2026-02-25

**Authors:** François Briand, Camille Le Cudennec, Estelle Grasset, Natalia Breyner, Claire Bigot, Pierre Dillard, Thierry Sulpice

**Affiliations:** ^1^ Physiogenex Escalquens France; ^2^ Janvier Labs Le Genest‐Saint‐Isle France

**Keywords:** hamster, MASH, mouse, obesity, retatrutide

## Abstract

**Objective/Methods:**

Our aim was to evaluate the efficacy of the triple glucagon, GIP, and GLP‐1 receptor agonist retatrutide in diet‐induced obese MASH mouse and hamster models, two preclinical models that we routinely use for assessing new therapies targeting obesity.

**Results:**

In mice, retatrutide strongly reduced body weight by 31% (*p* < 0.0001 vs. vehicle), both fat and lean mass, and food and water intake during the first days of treatment, while energy expenditure was not altered significantly. Retatrutide markedly reduced the HOMA‐IR index of insulin resistance, hepatic steatosis score, fatty acids, triglycerides, and total cholesterol content. In hamsters, retatrutide altered food preference with increased chow diet intake and decreased high fat/cholesterol diet and 10% fructose water intake. The significant weight loss was associated with a reduction in fat and lean mass, but the lean mass was not different after 5 weeks of treatment with no change in mineral bone density. Retatrutide significantly reduced HOMA‐IR, plasma triglycerides, and LDL‐cholesterol levels. Although retatrutide did not reduce histopathological scoring, there was a 50% reduction in hepatic triglyceride content (*p* < 0.01).

**Conclusions:**

Retatrutide demonstrates multiple metabolic benefits in both mouse and hamster models. Our preclinical setting will help to assess the efficacy of novel therapies targeting obesity and MASH.

## 1 Introduction

The prevalence of obesity and excessive body weight is continuously rising due to sedentary lifestyles and unhealthy diets. With billions of patients affected worldwide, this issue places a significant burden on public health systems in many countries [1, 2].

Indeed, obesity is characterized by a number of metabolic disturbances and comorbidities including, but not limited to, insulin resistance, type 2 diabetes, metabolic dysfunction‐associated steatohepatitis (MASH), and cardiometabolic complications [3], which in turn increases the cardiovascular risk for patients with obesity [4].

Fortunately, glucagon‐like peptide‐1 (GLP‐1) based therapies represent a unique opportunity for effectively treating obesity, leading to significant weight loss and additional benefits regarding obesity‐associated comorbidities [5]. Among those GLP‐1 based therapies, retatrutide, which acts as an agonist of the glucose‐dependent insulinotropic polypeptide (GIP), GLP‐1, and glucagon receptors, has demonstrated significant weight‐lowering effects, with a body weight change of up to 24% in a phase 2 clinical trial [6]. In patients with > 10% liver fat, retatrutide has also shown strong improvement in liver steatosis, with a change of up to 86% from baseline [7].

These positive results already obtained with GLP‐1 based therapies open new avenues for developing novel medications that could provide patients with even greater benefits [8]. To this end, animal models are needed to validate the efficacy of new agents and translate preclinical data to humans, thereby increasing the success rate of clinical trials. In the present study, our goal was to evaluate the efficacy of retatrutide in diet‐induced obese (DIO) MASH mouse and hamster models, two preclinical models we routinely use for assessing new therapies targeting obesity.

Although the DIO MASH mouse model is a routinely used model, it has several limitations, including a different lipid and lipoprotein metabolism compared to humans [9]. Meanwhile, we have recently developed a free choice DIO hamster model. Unlike the mouse and rat models, this preclinical model exhibits several human‐like features, including similar lipoprotein metabolism [10, 11], a similar bile acids profile [12], the presence of hepatocyte ballooning (a prerequisite for the MASH definition), and F2–F3 liver fibrosis [11, 12, 13]. We hypothesized that our hamster model would compensate for the limitations of the DIO MASH mouse model in order to evaluate the efficacy of retatrutide fully. Here, we present the metabolic benefits of retatrutide in both our B6 DIO MASH mice and our hamsters fed a free choice diet.

## 2 Methods

### 2.1 Animals and Diet

All animal protocols were reviewed and approved by the local (Comité régional d'éthique de Midi‐Pyrénées) and national (ministère de l'Enseignement Supérieur et de la Recherche) ethics committees (protocol number APAFIS#44475).

Male B6 DIO MASH mice, i.e., C57BL/6JRj, male, fed a modified AMLN‐diet (Research Diets reference #D09100310, 40% fat/20% fructose and 2% cholesterol) for 33 weeks were ordered from Janvier Labs and acclimatized for 2 weeks before the start of the experimental phase. Age‐matched male C57BL/6JRj mice (Janvier Labs) that were fed a control chow diet (5.1% fat, 19.3% protein, 55.5% carbohydrates, from Safe Diets) were used as negative/lean controls. After the acclimation period, animals were randomized based on their body weight, HOMA‐IR index (calculated from 6‐h fasting blood glucose and plasma insulin levels), and plasma transaminases (ALT and AST) levels. The mice were then treated subcutaneously every 3 days for 5 weeks with vehicle or retatrutide 15 nmol/kg. This dose was selected based on the publication of Coskun et al. [14]. Lean mice on chow diet were also treated with vehicle subcutaneously every 3 days. During the 5‐week treatment period, body composition was assessed at 2 weeks and at the end of treatment using a Minispec (Bruker), which acquires and analyzes time domain‐NMR signals from all protons in the entire body to deliver three components of interest: fat, free body fluid, and lean tissue values. Food intake, water intake, and energy expenditure were assessed in metabolic cages using the Phenomaster system (TSE system). Energy expenditure was analyzed based on the consensus guide to preclinical indirect calorimetry experiments [15], to compare DIO MASH mice treated with vehicle or retatrutide using ANCOVA made available on a free online resource (https://www.mmpc.org/shared/regression.aspx). At the end of the treatment period, mice were fasted for 6 h, and blood was collected on heparin tubes for biochemistry and, after exsanguination with saline, the liver was collected for biochemistry and histology analysis.

Male golden Syrian hamsters (Janvier Labs), 4 weeks old at the beginning of the study, were acclimatized for 5 days before being fed for a minimum of 20 weeks with a free choice diet to induce obesity and MASH. This diet was selected to evaluate the effects of drugs on food preference, while a single high‐fat diet does not induce obesity in hamsters [11]. As described previously [11, 12, 13], the free choice diet consists of a choice, within the same cage, between control diet with normal water or a high‐fat/high‐cholesterol diet (40.8% fat, 14.8% protein, 44.4% carbohydrates, and 0.5% cholesterol from Safe Diets) with 10% fructose‐enriched drinking water. The high‐fat/high‐cholesterol diet was a mixture of 55% control chow diet, 20% peanut butter paste (Skippy, Hormel Foods Corp.) and 25% hazelnut paste (Nustikao, Leclerc), with vegetable oils as fat source, as previously described [11]. As with the mice, hamsters were randomized into two treatment groups after the free choice diet‐induction period and were then treated subcutaneously every 3 days for 5 weeks with vehicle or retatrutide 15 nmol/kg. This dose was selected based on previous unpublished studies performed by Physiogenex. Body composition was assessed using a Minispec (Bruker) at Day 4, Day 13, and Day 31 of treatment. As for B6 DIO MASH mice, blood and liver samples were collected at the end of the 5‐week treatment period for biochemistry and histology analysis.

### 2.2 Biochemical Analysis

Plasma biochemistry was performed by the Genotoul Anexplo platform in Toulouse, France. Plasma ALT and AST, total cholesterol, HDL‐cholesterol, LDL‐cholesterol, free fatty acids, and triglycerides were determined using a Horiba Pentra 400 machine and related Pentra assay kits (Horiba France SAS). Fast protein liquid chromatography (FPLC) analysis (total cholesterol and triglycerides) was performed as described previously [11]. Colorimetric assay kits were used to assay hepatic total cholesterol, triglycerides, and fatty acids (references # WCHO100, WTRIG1000, and W1W434‐91795 from Sobioda, Montbonnot‐Saint‐Martin, France) from liver homogenate after lipid solubilization with deoxycholate, as previously described [11].

### 2.3 Histology Analysis

Liver histology analysis was performed by Dr. Virgile Richard and his team at Sciempath using hematoxylin & eosin (H&E) and Sirius red staining for histopathological scoring and %Sirius red labeling, as previously described [11]. As reported by others [16], hepatocyte ballooning is not observed in diet‐induced MASH mouse models and therefore was not scored in the present B6 DIO MASH mouse model.

### 2.4 Bone Mineral Density Measurements

Micro‐CT analyses were performed at proximal and diaphyseal levels of hamsters' tibias to evaluate both the trabecular and cortical bone, respectively, by Atlantic Bone Screen, Nantes, France, as described previously [17].

### 2.5 Gene Expression Analysis

Gene expression analysis was performed using real time PCR by Mr. Thierry Leste‐Lasserre at the Neurocentre Magendie in Bordeaux, France, as previously described [11]. Primers sequences are reported in Table S1 for mice and Table S2 for hamsters.

### 2.6 Statistical Analysis

Data are presented as mean ± SEM. Ordinal data (i.e., steatosis, inflammation, ballooning, and fibrosis scores) are presented as median. Unpaired two‐tailed Student's *t*‐test, Mann–Whitney, or two‐way ANOVA + Bonferroni posttest was used for statistical analysis using GraphPad Prism software. A *p* value < 0.05 was considered significant.

## 3 Results

### 3.1 Retatrutide Strongly Reduces Body Weight With Significant Reduction in Fat and Lean Mass and Food Intake but Unchanged Energy Expenditure in B6 DIO MASH Mice

**FIGURE 1:**
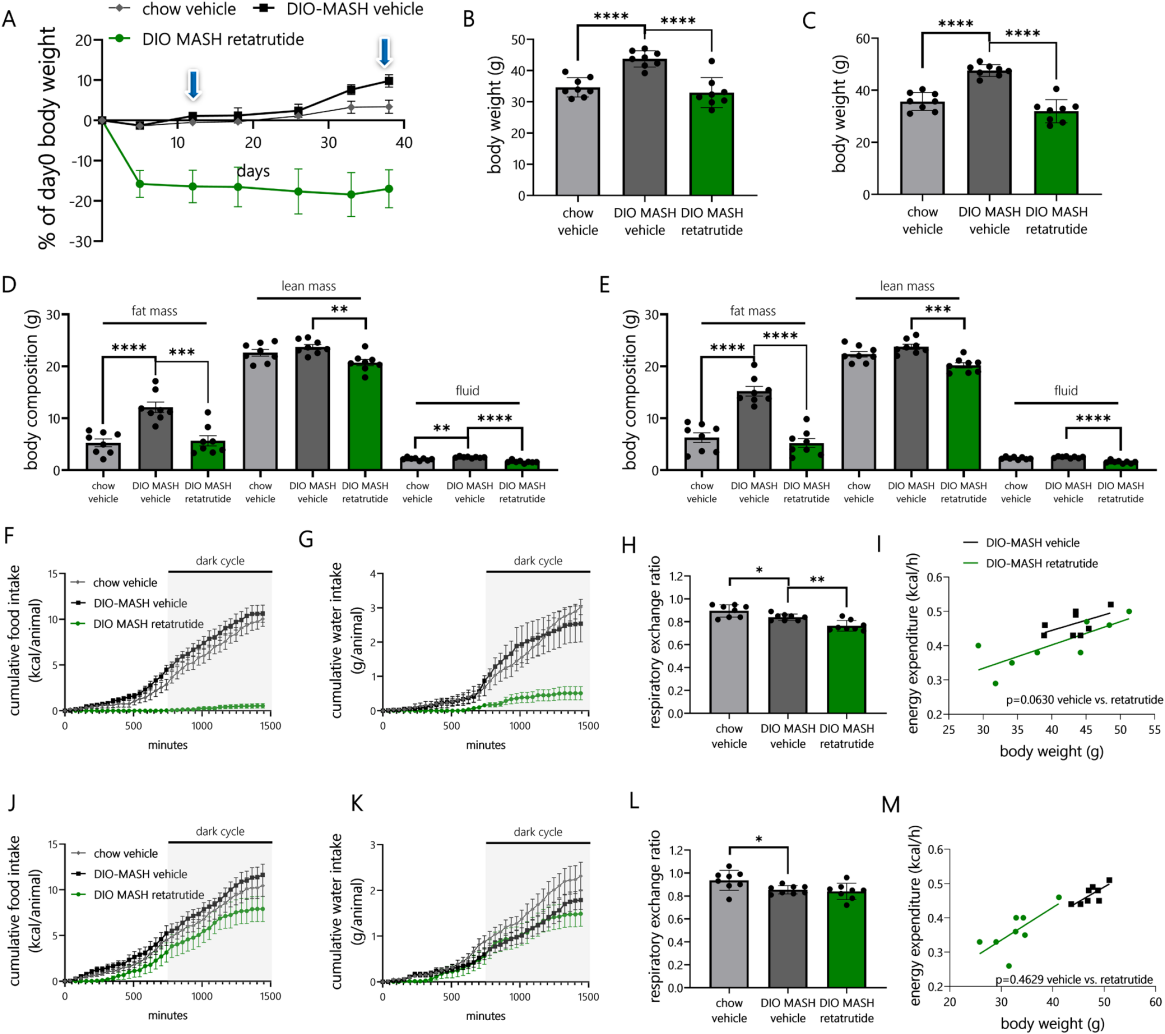
Retatrutide reduces body weight with changes in body composition and food intake but not energy expenditure in B6 DIO MASH mice. (A) Body weight change as % baseline. Blue arrows indicate 2‐week and 5‐week time points when body composition was assessed. Body weight at (B) 2 weeks and (C) end of the 5‐week treatment. Body composition at (D) 2 weeks and (E) end of the 5‐week treatment. (F) Food intake, (G) water intake, (H) respiratory exchange ratio, and (I) energy expenditure at Day 3 of treatment. (J) Food intake, (K) water intake, (L) respiratory exchange ratio, and (M) energy expenditure at the end of the 5‐week treatment, in chow fed mice treated with vehicle and B6 DIO MASH mice treated with vehicle or retatrutide. Data are shown as mean ± SEM; *n* = 8/group; *p < 0.05, **p < 0.01, ***p < 0.001, and ****p < 0.0001. [Color figure can be viewed at wileyonlinelibrary.com]

While vehicle‐treated B6 DIO MASH mice showed a trend toward higher body weight, those treated with retatrutide showed a rapid and substantial weight loss that sustained until the end of the treatment period (Figure 1A). After 2 weeks of treatment, B6 DIO MASH mice had a 26% higher body weight than chow fed mice treated with vehicle (*p* < 0.0001). Retatrutide treatment led to a 25% reduction in body weight (*p* < 0.0001 vs. vehicle), which was similar to the values observed in chow fed mice (Figure 1B). These significant differences in body weight were maintained at the end of the 5‐week treatment period (Figure 1C). Body composition was assessed at 2 weeks of treatment (Figure 1D) and at the end of treatment (Figure 1E). As expected, B6 DIO MASH mice treated with vehicle had significantly higher fat mass when compared with mice fed the chow diet (*p* < 0.0001 at both time points). In contrast, there was no difference in lean mass between the two groups and body fluid values were similar, although they were significantly higher at the 2‐week time point for B6 DIO MASH mice treated with vehicle. Consistent with its substantial weight‐lowering effect, treatment with retatrutide induced a significant reduction in fat mass (−53% at 2 weeks and −65% at the end of the treatment period). This weight loss also induced a significant reduction in lean mass (−13% at 2 weeks and −15% by the end of the treatment). Significant reductions in body fluid were also observed at both time points. It is worth noting that different trends were observed when body fat and lean and fluid mass were expressed as a percentage of body weight at the end of the treatment (Table 1). Although % fat mass was significantly higher in B6 DIO MASH mice treated with vehicle and significantly reduced by retatrutide treatment, B6 DIO MASH mice had a significantly lower % lean mass than chow fed mice, and retatrutide treatment resulted in a significantly higher % lean mass, as compared with B6 DIO MASH mice treated with vehicle.

**TABLE 1:** Clinical and biochemical parameters after 5 weeks of treatment with vehicle or retatrutide in chow diet fed and B6 DIO MASH mice.

Parameter	Chow vehicle	B6 DIO MASH vehicle	B6 DIO MASH retatrutide
Body fat mass (% body weight)	17.2 ± 2.2	31.8 ± 1.5****	15.7 ± 1.9^####^
Body lean mass (% body weight)	63.0 ± 2.1	50.0 ± 1.3***	63.7 ± 1.7^####^
Body fluid mass (% body weight)	6.5 ± 0.2	5.3 ± 0.1****	4.9 ± 0.1
Fasting blood glucose (mg/dL)	146 ± 8	189 ± 14*	139 ± 4^###^
Plasma insulin (μU/mL)	41 ± 8	45 ± 4	19 ± 4^###^
HOMA‐IR index ([mM × μU/mL]/22.5)	16 ± 3	21 ± 3	7 ± 1^###^
Plasma free fatty acids (mM)	1.21 ± 0.08	1.22 ± 0.06	1.00 ± 0.08
Plasma triglycerides (g/L)	0.89 ± 0.06	0.36 ± 0.02****	0.39 ± 0.04
Plasma total cholesterol (g/L)	0.91 ± 0.03	2.42 ± 0.23****	1.52 ± 0.09^##^
Plasma ALT (U/L)	177 ± 58	440 ± 92*	318 ± 69
Plasma AST (U/L)	170 ± 24	465 ± 86*	621 ± 111
Liver weight (g)	1.44 ± 0.04	3.50 ± 0.22***	1.96 ± 0.15^###^
Hepatic fatty acids (μmol/g liver)	10.2 ± 1.1	72.8 ± 2.2****	24.1 ± 1.3^####^
Hepatic triglycerides (mg/g liver)	25.1 ± 3.0	236.5 ± 15.4****	58.8 ± 5.5^###^
Hepatic total cholesterol (mg/g liver)	2.2 ± 0.1	18.9 ± 1.6***	10.4 ± 0.8^###^

*Note*: Data are shown as mean ± SEM, *n* = 8 mice per group.

*p < 0.05, ***p < 0.001, ****p < 0.0001, chow vehicle versus B6 DIO MASH vehicle; ##*p* < 0.01, ###*p* < 0.001, #### *p* < 0.0001, B6 DIO MASH vehicle versus B6 DIO MASH retatrutide. Statistical analysis was performed using an unpaired two‐tailed Student *t*‐test or a Mann–Whitney test.

Mice were also placed in metabolic cages to monitor food and water intake, as well as energy expenditure at the beginning (Day 3) and the end of the treatment. After only two subcutaneous doses of retatrutide (Day 3 of treatment), food intake was strongly inhibited (Figure 1F), and water intake was very low (Figure 1G). Compared to chow fed mice, respiratory exchange ratio (Figure 1H) was significantly lower in DIO MASH mice treated with vehicle, and the ratio was further reduced in DIO MASH mice treated with retatrutide (*p* < 0.01 vs. vehicle). ANCOVA showed a trend toward lower energy expenditure with retatrutide, but this was not significant (Figure 1I). At the end of the treatment, the food intake‐lowering effect of retatrutide was still evident, albeit less pronounced (Figure 1J), while water intake approached the levels observed in the vehicle group (Figure 1K). There was no difference in respiratory exchange ratio (Figure 1L) and energy expenditure (Figure 1M) between DIO MASH mice treated with vehicle or retatrutide.

Altogether, these data indicate that retatrutide strongly reduces body weight with significant reductions in fat and lean mass and food and water intake but unchanged energy expenditure in B6 DIO MASH mice.

### 3.2 Retatrutide Lowers HOMA‐IR Index of Insulin Resistance and Substantially Reduces Liver Weight and Lipid Content in B6 DIO MASH Mice

Plasma and liver biochemical parameters were measured at the end of the 5‐week treatment period (Table 1). Compared to chow fed mice treated with vehicle, B6 DIO MASH mice treated with vehicle tended to show a higher HOMA‐IR index of insulin resistance, due to higher fasting blood glucose levels (+29%, *p* < 0.05 vs. chow), despite similar plasma insulin levels. In contrast, retatrutide treatment in B6 DIO MASH mice strongly lowered HOMA‐IR index mean value by 69% (*p* < 0.001 vs. vehicle). This reduction in the HOMA‐IR index was due to a significant reduction in both fasting blood glucose (−27%) and plasma insulin (−57%) levels. Plasma free fatty acid levels were not different between chow fed mice and B6 DIO MASH mice treated with vehicle. Compared with B6 DIO MASH mice treated with vehicle, retatrutide tended to lower plasma free fatty acid levels, but this did not reach statistical significance (*p* = 0.0565 vs. vehicle). As expected in this DIO MASH mouse model, plasma triglyceride levels (Table 1) were significantly lower than the levels observed in chow fed mice (−60%, *p* < 0.0001). Hence, any triglyceride‐lowering effect of retatrutide could not be detected and plasma triglyceride levels remained similar to the mean values observed in the vehicle group. Plasma total cholesterol levels were higher in B6 DIO MASH mice as compared to chow fed mice (+167%, *p* < 0.0001), and retatrutide treatment led to a 37% reduction, as compared with B6 DIO MASH treated with vehicle (*p* < 0.01).

As expected, B6 DIO MASH mice had higher liver weight and lipid content, as compared to chow fed mice (all *p* < 0.0001). Treatment with retatrutide in B6 DIO MASH mice led to a marked reduction in liver weight (−44%, *p* < 0.0001 vs. vehicle). This liver weight‐lowering effect was associated with significant reductions in hepatic fatty acids, triglycerides, and total cholesterol levels (−69%, −75%, and −45%, respectively).

Taken together, these data demonstrate that the weight‐lowering effect of retatrutide induced additional benefits including improved insulin resistance, lower cholesterolemia, and a strong reduction in liver weight and hepatic lipid content in B6 DIO MASH mice.

### 3.3 A 5‐Week Treatment With Retatrutide Improves Liver Steatosis but Not Inflammation and Fibrosis in B6 DIO MASH Mice

**FIGURE 2:**
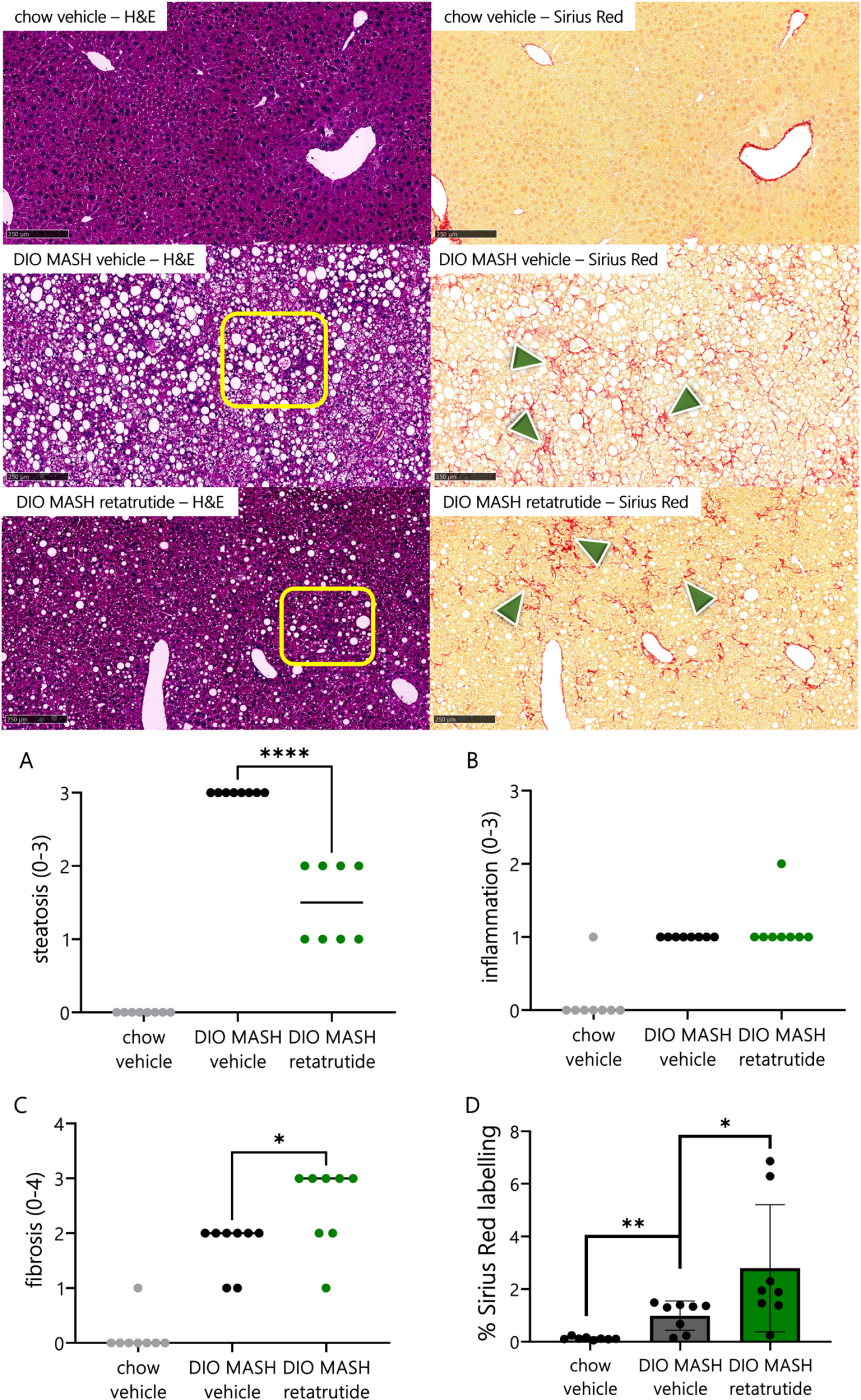
A 5‐week treatment with retatrutide improves liver steatosis but not inflammation and fibrosis in B6 DIO MASH mice. Upper panel shows representative H&E and Sirius red staining in chow fed mice treated with vehicle and B6 DIO MASH mice treated with vehicle or retatrutide. Yellow square indicates steatosis and inflammatory foci; green arrows indicate fibrosis. (A) Hepatic steatosis, (B) inflammation, (C) fibrosis, and (D) %Sirius red labeling at the end of the 5‐week treatment period, in chow fed mice treated with vehicle and B6 DIO MASH mice treated with vehicle or retatrutide. Data are shown as mean ± SEM; *n* = 8/group; *p < 0.05, **p < 0.01, and ****p < 0.0001. [Color figure can be viewed at wileyonlinelibrary.com]

Histological analysis (representative images in Figure 2) was used to perform histopathological scoring. As expected, mice fed a chow diet had no liver lesions while B6 DIO MASH mice treated with vehicle showed a maximal liver steatosis score (Figure 2A), a low inflammation score of 1 (Figure 2B), and perisinusoidal (score 1) to portal (score 2) fibrosis (Figure 2C). Accordingly, liver %Sirius red labeling was significantly higher in B6 DIO MASH mice, indicating higher collagen content (Figure 2D). In line with the substantial reduction in hepatic lipid content, treatment with retatrutide led to a substantial reduction in liver steatosis score (*p* < 0.0001 vs. vehicle). However, it had a neutral effect on the inflammation score and significantly raised the fibrosis score with few individuals reaching a bridging fibrosis score of 3. Consistent with this outcome, retatrutide treatment resulted in significantly higher %Sirius red labeling as compared to B6 DIO MASH mice treated with vehicle (Figure 2D).

To further investigate the effects of retatrutide on liver lesions, the expression of genes involved in liver lipogenesis, inflammation, and fibrosis was assessed by RT‐qPCR (Table 2). Compared to chow fed mice treated with vehicle, B6 DIO MASH mice treated with vehicle showed higher expression for genes involved in lipogenesis such as Srebf1, Scd1, and Acc. However, the expression of Acly, which has a central role in Acyl‐CoA synthesis, and thus lipids and cholesterol synthesis, was significantly lower. B6 DIO MASH mice treated with vehicle also had lower expression of the proinflammatory cytokines IL‐6 and IL‐1β, but not TNF‐α, which was significantly higher as compared to chow fed mice. Additionally, CCl2, which encodes for macrophage‐chemoattractant protein‐1, and Adrge1, which encodes for F4/80 (a marker of macrophages in mice), showed higher expression versus chow fed mice. Additionally, the hepatic expression of profibrotic genes (Acta2, Timp1, Col1α1, and Tgfβ1) was significantly higher in B6 DIO MASH mice treated with vehicle. Although retatrutide did not reduce the expression of genes involved in lipogenesis, the triple agonist reduced the expression of genes encoding for proinflammatory cytokines (TNF‐α and Adrge1) and profibrotic markers (Acta2, Timp1, and Col1α1).

**TABLE 2:** Hepatic gene expression after 5 weeks of treatment with vehicle or retatrutide in chow diet fed and B6 DIO MASH mice.

Hepatic gene expression (fold‐change vs. chow vehicle)	Chow vehicle	B6 DIO MASH vehicle	B6 DIO MASH retatrutide
Srebf1	1.00 ± 0.09	2.97 ± 0.22***	3.27 ± 0.31
Scd1	1.04 ± 0.11	2.19 ± 0.18****	2.22 ± 0.35
Acca	1.00 ± 0.06	1.51 ± 0.12**	2.19 ± 0.34
Acly	1.00 ± 0.11	0.60 ± 0.07*	1.39 ± 0.30
Cpt1a	1.00 ± 0.11	1.22 ± 0.04	0.92 ± 0.09
Il‐6	1.00 ± 0.15	0.79 ± 0.23	0.41 ± 0.07
Il‐1b	1.00 ± 0.11	0.65 ± 0.05*	0.38 ± 0.05^##^
Tnf‐alpha	1.00 ± 0.28	5.49 ± 0.69***	3.46 ± 0.55^#^
CCl2	1.00 ± 0.12	18.61 ± 8.60**	7.75 ± 0.98
Adgre1	1.00 ± 0.12	2.07 ± 0.21***	1.53 ± 0.13^#^
Acta2	1.00 ± 0.05	1.73 ± 0.10****	1.31 ± 0.12^#^
Timp1	1.00 ± 0.38	23.60 ± 4.53***	8.73 ± 1.51^##^
Col1a1	1.00 ± 0.22	13.38 ± 2.39***	5.63 ± 0.77^#^
Tgfb1	1.00 ± 0.08	2.08 ± 0.22***	1.72 ± 0.12

*Note*: Data are shown as mean ± SEM, *n* = 8 mice per group.

*p < 0.05, **p < 0.01, ***p < 0.001, ****p < 0.0001, chow vehicle versus B6 DIO MASH vehicle; #*p* < 0.05, ## *p* < 0.01, B6 DIO MASH vehicle versus B6 DIO MASH retatrutide. Statistical analysis was performed using an unpaired two‐tailed Student *t*‐test or a Mann–Whitney test.

Overall, a 5‐week treatment with retatrutide in B6 DIO MASH did not improve liver lesions but gene expression data suggest that the triple agonist induced anti‐inflammatory and antifibrotic mechanisms that could potentially lead to benefits with longer‐term treatment.

### 3.4 In Free Choice Diet Fed Hamsters, Retatrutide Induces Changes in Food Preference, Sustained Fat Mass Reduction but Transient Lean Mass Loss, and Unaltered Bone Density

**FIGURE 3:**
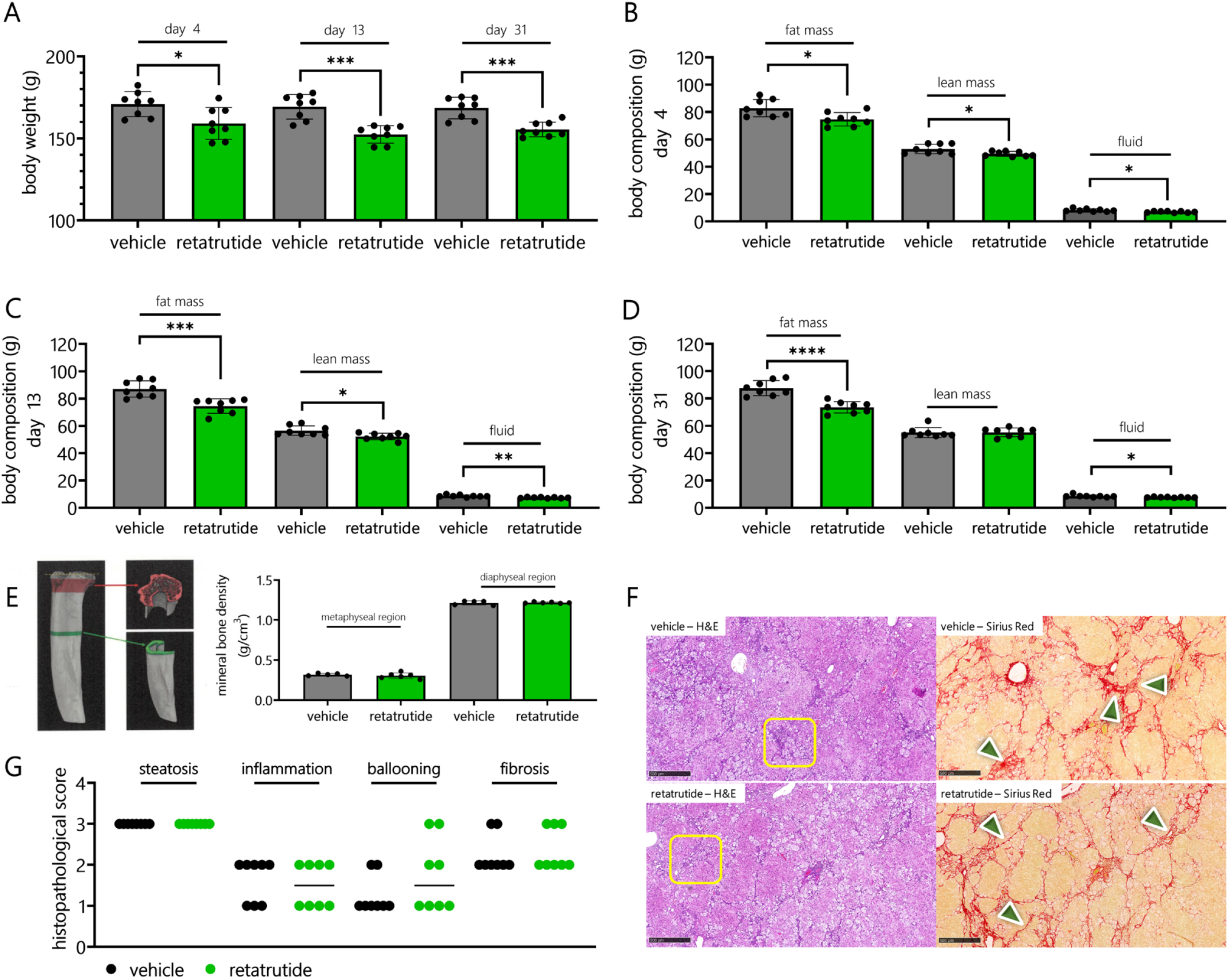
Retatrutide reduces body weight with changes in body composition but unchanged mineral bone density and liver lesions in the free choice diet fed hamster model. (A) Body weight on Day 4, Day 13, and Day 31. Body composition at (B) Day 4, (C) Day 13, and (D) Day 31. (E) Mineral bone density of the metaphyseal (red) and diaphyseal (green) volume of interest in the tibia by micro‐CT. (F) Representative H&E and Sirius red pictures and (G) histopathological scoring in free choice diet fed hamsters treated with vehicle or retatrutide. Data are shown as mean ± SEM; *n* = 8/group; *p < 0.05, **p < 0.01, and ***p < 0.001. [Color figure can be viewed at wileyonlinelibrary.com]

A 5‐week treatment with retatrutide was next evaluated in the free choice DIO hamster model. Similar to the results observed in B6 DIO MASH mice, retatrutide led to a significant reduction in body weight already on Day 4 of treatment and similar reductions were observed on Day 13 and Day 31 of treatment (Figure 3A). As was previously reported for the GLP‐1 receptor agonist semaglutide in this model [13], the weight‐lowering effect of retatrutide was associated with an altered food preference, i.e., higher intake of the chow diet, reduced intake of the high‐fat diet, unchanged normal water intake, and a trend toward lower fructose‐enriched water intake (Table 3).

**TABLE 3:** Clinical and biochemical parameters after 5 weeks of treatment with vehicle or retatrutide in free choice diet fed hamsters.

Parameter	Vehicle	Retatrutide
Body fat mass (% body weight)	52.0 ± 0.7	47.3 ± 0.7***
Body lean mass (% body weight)	32.7 ± 0.6	35.5 ± 0.7*
Body fluid mass (% body weight)	5.1 ± 0.2	5.0 ± 0.1
5‐week cumulative chow diet intake (g/animal)	49.9 ± 3.6	85.5 ± 7.2*
5‐week cumulative high‐fat diet intake (g/animal)	128.4 ± 3.3	72.1 ± 2.5***
5‐week cumulative water intake (mL/animal)	318.3 ± 15.2	290.5 ± 30.8
5‐week cumulative 10% fructose water intake (mL/animal)	151.6 ± 14.4	132.2 ± 6.3
Fasting blood glucose (mg/dL)	79 ± 5	65 ± 3*
Plasma insulin (μU/mL)	18.2 ± 2.4	4.3 ± 0.5***
HOMA‐IR index ([mM × μU/mL]/22.5)	3.5 ± 0.5	0.7 ± 0.1***
Plasma free fatty acids (mM)	0.69 ± 0.05	0.53 ± 0.04*
Plasma triglycerides (g/L)	1.32 ± 0.11	0.91 ± 0.10*
Plasma total cholesterol (g/L)	4.23 ± 0.27	3.32 ± 0.16*
Plasma LDL‐cholesterol (g/L)	0.96 ± 0.12	0.55 ± 0.08*
Plasma HDL‐cholesterol (g/L)	2.45 ± 0.20	2.50 ± 0.11
Plasma ALT (U/L)	434 ± 123	282 ± 50
Plasma AST (U/L)	171 ± 52	113 ± 22
Liver weight (g)	13.9 ± 0.4	11.7 ± 0.3*
Hepatic fatty acids (μmol/g liver)	53.3 ± 2.6	51.3 ± 2.6
Hepatic triglycerides (mg/g liver)	11.5 ± 1.6	5.7 ± 1.0*
Hepatic total cholesterol (mg/g liver)	102.7 ± 5.4	84.2 ± 11.1

*Note*: Data are shown as mean ± SEM, *n* = 8 hamsters per group.

*p < 0.05, ***p < 0.001, vehicle versus retatrutide. Statistical analysis was performed using an unpaired two‐tailed Student *t*‐test or a Mann–Whitney test.

Body composition was also monitored on Day 4, Day 13, and Day 31 (Figure 3B–D). As was observed in mice, hamsters treated with retatrutide showed a significant reduction in fat mass and body fluid at all time points. A significant reduction in lean mass was also observed, but this appeared limited on Day 4 and 13, with no difference observed on Day 31. Indeed, when body composition was expressed as percentage of body weight on Day 31, fat mass was significantly lower, but lean mass was significantly higher with retatrutide treatment (Table 3).

We also investigated whether changes in body weight and composition could affect bone density. However, mineral bone density of the metaphyseal and diaphyseal regions assessed by micro‐CT did not differ between the vehicle‐ and retatrutide‐treated groups (Figure 3E).

As was observed in mice, these data indicate that retatrutide also induces weight loss alongside changes in body composition but has limited effects on lean mass and bone density.

### 3.5 In Hamsters, a 5‐Week Treatment With Retatrutide Significantly Improves Insulin Resistance, Dyslipidemia, and Liver Triglyceride Content

Compared with vehicle, retatrutide significantly reduced fasting blood glucose (−20%) and plasma insulin (−76%) levels (Table 3). This led to an 80% lower HOMA‐IR index of insulin resistance (*p* < 0.001 vs. vehicle). Retatrutide also improved dyslipidemia with a significant reduction in plasma triglyceride levels (−31% vs. vehicle) and total cholesterol levels (−21%). Interestingly, retatrutide significantly reduced LDL‐cholesterol levels (−43% vs. vehicle) but did not alter HDL‐cholesterol levels. This beneficial effect on dyslipidemia was further confirmed by FPLC analysis (Figure S1).

Compared with vehicle treatment, plasma ALT and AST levels were reduced by 35% and 34%, respectively, in hamsters treated with retatrutide; however, this reduction was not statistically significant. As observed in mice, retatrutide significantly reduced liver weight (−14%, *p* < 0.05 vs. vehicle), albeit less substantially. There was also no alteration of hepatic fatty acids and total cholesterol levels, but liver triglyceride content was significantly reduced by 50% as compared to vehicle.

Overall, these data demonstrate that retatrutide improves insulin resistance, dyslipidemia, and liver triglyceride levels in free choice diet fed hamsters.

### 3.6 A 5‐Week Treatment With Retatrutide Does Not Alter Liver Lesions in Free Choice Diet Fed Hamsters but Significantly Reduces Hepatic Expression of Proinflammatory and Profibrotic Genes

Histological analysis (representative pictures in Figure 3F) was used to perform histopathological scoring. Compared with vehicle, retatrutide had no significant effect on the scores for steatosis, inflammation, hepatocyte ballooning, and fibrosis (Figure 3G). Additionally, there was no difference in the %Sirius red labeling (vehicle: 0.45% ± 0.12%; retatrutide: 0.49% ± 0.10%). To further investigate the effects of retatrutide on liver lesions, the expression of genes involved in liver lipid/lipoprotein metabolism, inflammation, and fibrosis was assessed by RT‐qPCR (Table 4). Retatrutide had no effect on the hepatic expression of Fas, Acca, Cpt1a, SR‐BI, LDL‐r, and Cyp7a1 (data not shown). However, retatrutide significantly reduced the expression of genes coding for the proinflammatory cytokines IL‐1β, IL‐6, and CCl2 (all *p* < 0.05 vs. vehicle). A significant reduction in the gene expression of the proapoptotic caspase 3 was also observed. Although the trend was not significant for Acta2, retatrutide demonstrated a significant lowering effect on the expression of the profibrotic genes Col1a1, Col3a1, and Timp1.

**TABLE 4:** Hepatic gene expression after 5 weeks of treatment with vehicle or retatrutide in free choice diet fed hamsters.

Hepatic gene expression (fold‐change vs. vehicle)	Vehicle	Retatrutide
Fas	1.00 ± 0.12	0.76 ± 0.11
Cpt1a	1.00 ± 0.09	1.10 ± 0.18
Acca	1.00 ± 0.10	1.16 ± 0.07
Scarb1	1.00 ± 0.12	1.17 ± 0.16
LDL‐r	1.00 ± 0.05	1.13 ± 0.17
Cyp7a1	1.00 ± 0.44	0.69 ± 0.18
Il‐1b	1.00 ± 0.21	0.53 ± 0.09*
Il‐6	1.00 ± 0.25	0.44 ± 0.10*
CCl2	1.00 ± 0.21	0.44 ± 0.11*
Casp3	1.00 ± 0.07	0.75 ± 0.04*
Acta2	1.00 ± 0.14	0.79 ± 0.13
Timp1	1.00 ± 0.35	0.29 ± 0.06*
Col1a1	1.00 ± 0.33	0.24 ± 0.05**
Col3a1	1.00 ± 0.17	0.45 ± 0.07*

*Note*: Data are shown as mean ± SEM, *n* = 6 hamsters per group.

*p < 0.05, **p < 0.01, vehicle versus retatrutide. Statistical analysis was performed using an unpaired two‐tailed Student *t*‐test or a Mann–Whitney test.

Taken together, these data indicate that a 5‐week treatment with retatrutide did not lead to significant changes to liver lesions; however, it significantly reduced the expression of proinflammatory and profibrotic genes.

## 4 Discussion

As demonstrated in humans, the GIP/GLP‐1/glucagon receptor triple agonist retatrutide had evident weight‐lowering effects in both our B6 DIO MASH mouse model and our free choice diet fed hamster model. In parallel with the reduction in food intake, the unchanged energy expenditure contributed to the lower body weight maintenance, as described by others in DIO mice [14]. With the very dynamic development of GLP‐1 based therapies, a rising concern regarding the potential loss of muscle and bone with weight loss has emerged [18]. Whether the absolute lean mass reduction is actually related to a loss in muscle mass remains to be confirmed. Indeed, the lean mass also includes bone and organs (e.g., liver), so a change in this parameter does not necessarily indicate a change in muscle mass alone [19]. While bone density remained unchanged in hamsters, the lean mass expressed as percentage of body weight was higher in both mice and hamsters after 5 weeks of treatment with retatrutide. Therefore, retatrutide's weight‐lowering effect mainly targets fat mass rather than lean mass, which may contribute to the metabolic benefits observed in both models.

In the B6 DIO MASH mouse model, the 5‐week treatment with retatrutide strongly reduced liver fat content, as observed in a human phase 2a trial where liver fat was reduced by up to 82% [7]. However, this did not lead to any improvement in liver inflammation and fibrosis, and few individual mice even showed a worsening of liver scar tissue with bridging fibrosis. Although it remains to be investigated, very rapid fat mobilization and fatty acids oxidation occurring during weight loss could promote oxidative stress, inflammation, and in turn liver fibrosis [20]. The detrimental effects of rapid weight loss have been reported in a MASLD rat model and in humans following bariatric surgery [21, 22, 23]. Therefore, a longer treatment period with retatrutide may be needed to observe a reduction in liver fibrosis. The significant reduction in the hepatic expression of genes involved in inflammation and fibrogenesis would support this hypothesis. A similar reduction in the expression of genes involved in inflammation and fibrosis has been reported in a 31‐day MASH mouse model. However, this study did not present any histology data [24]. Activation of the hepatic glucagon receptor by retatrutide is expected to stimulate lipolysis and fatty acid oxidation [25], which would prevent or reduce liver fibrosis in the long term. In a nonobese rodent model of fibrotic MASH, the GIP/GLP‐1/glucagon receptor triple agonist HM15211 also demonstrated antifibrotic effects that remain to be confirmed in humans [26]. Additionally, a GLP‐1/glucagon receptor dual agonist also demonstrated antifibrotic effects in a CCl4‐induced liver fibrosis mouse model [27]. Therefore, with an extended treatment period, retatrutide could potentially block proinflammatory and hepatocyte apoptosis pathways to prevent fibrogenesis, but this remains to be demonstrated in both preclinical studies and human clinical trials.

In the free choice DIO MASH hamster model, retatrutide had limited hepatic effects, i.e., reducing triglyceride content without exacerbating liver lesions. Similar effects were observed in the same model with the GLP‐1 receptor agonist semaglutide [13]. Within the 5‐week treatment window, adding both GIP and glucagon receptor agonism with retatrutide did not demonstrate any additional benefit compared to GLP‐1 agonism alone (i.e., compared to semaglutide treatment). Given the lower hepatic expression of proinflammatory and profibrotic genes observed in the hamster, extending the retatrutide treatment duration may be necessary to detect potential benefits in MASH and liver fibrosis with the triple agonist. Additionally, retatrutide demonstrated triglyceride‐ and cholesterol‐lowering effects, including a significant reduction in LDL‐cholesterol levels. As expected in this model showing a human‐like lipoprotein metabolism [10], these effects align with data collected from human patients with obesity, in whom retatrutide was shown to significantly reduce plasma triglyceride and non‐HDL‐cholesterol levels [28]. As previously observed with semaglutide [13], retatrutide positively altered the food preference of hamsters for high‐fat diet and fructose‐enriched water. This also highlights the potential of this triple agonist to also improve eating behavior disorders in humans, which would also contribute to the long‐term weight‐lowering effects and metabolic benefits.

Taken together, the present preclinical data on retatrutide emphasize the importance of combining different rodent models to better predict the benefits of novel therapies targeting obesity and associated comorbidities.

## 5 Conclusion

Using the B6 DIO MASH mouse and the free choice diet fed hamster, we have demonstrated the multiple metabolic benefits on one hand, and on the other hand, the limited effects on MASH/liver fibrosis of a 5‐week treatment with retatrutide. However, the lower hepatic expression of genes involved in inflammation and fibrosis suggested that longer treatment could result in improved liver histology in both models.

## Author Contributions

François Briand, Camille Le Cudennec, Pierre Dillard, and Thierry Sulpice conceived the study. François Briand analyzed and interpreted the data and drafted the manuscript. Natalia Breyner and Claire Bigot performed the analysis of energy expenditure data using ANCOVA. Estelle Grasset, Natalia Breyner, Claire Bigot, and all other authors read and critically reviewed and edited the manuscript.

## Conflicts of Interest

François Briand, Estelle Grasset, Claire Bigot, Natalia Breyner, and Thierry Sulpice are employees of Physiogenex. François Briand and Thierry Sulpice have shares in Physiogenex. Pierre Dillard and Camille Le Cudennec are employees of Janvier Labs, France.

## Supporting information


**Figure S1:** Retatrutide improves dyslipidemia in free choice diet fed hamsters. (A) total cholesterol and (B) triglycerides lipoprotein profiles measured by fast protein liquid chromatography at the end of the 5‐week treatment with vehicle or retatrutide in free choice diet fed hamsters.

**Table S1:** Mouse qPCR primer sequences.

**Table S2:** Hamster qPCR primer sequences

## Data Availability

The data that support the findings of this study are available from the corresponding author upon reasonable request.
