# Supplementary figures and images for: Retatrutide Shows Multiple Metabolic Benefits in Diet‐Induced Obese MASH Mouse and Hamster Models
Source: Obesity (Silver Spring). 2026 Feb 25;34(Suppl 1):43–53. doi: 10.1002/oby.70155 (PMC13250738; doi:10.1002/oby.70155)

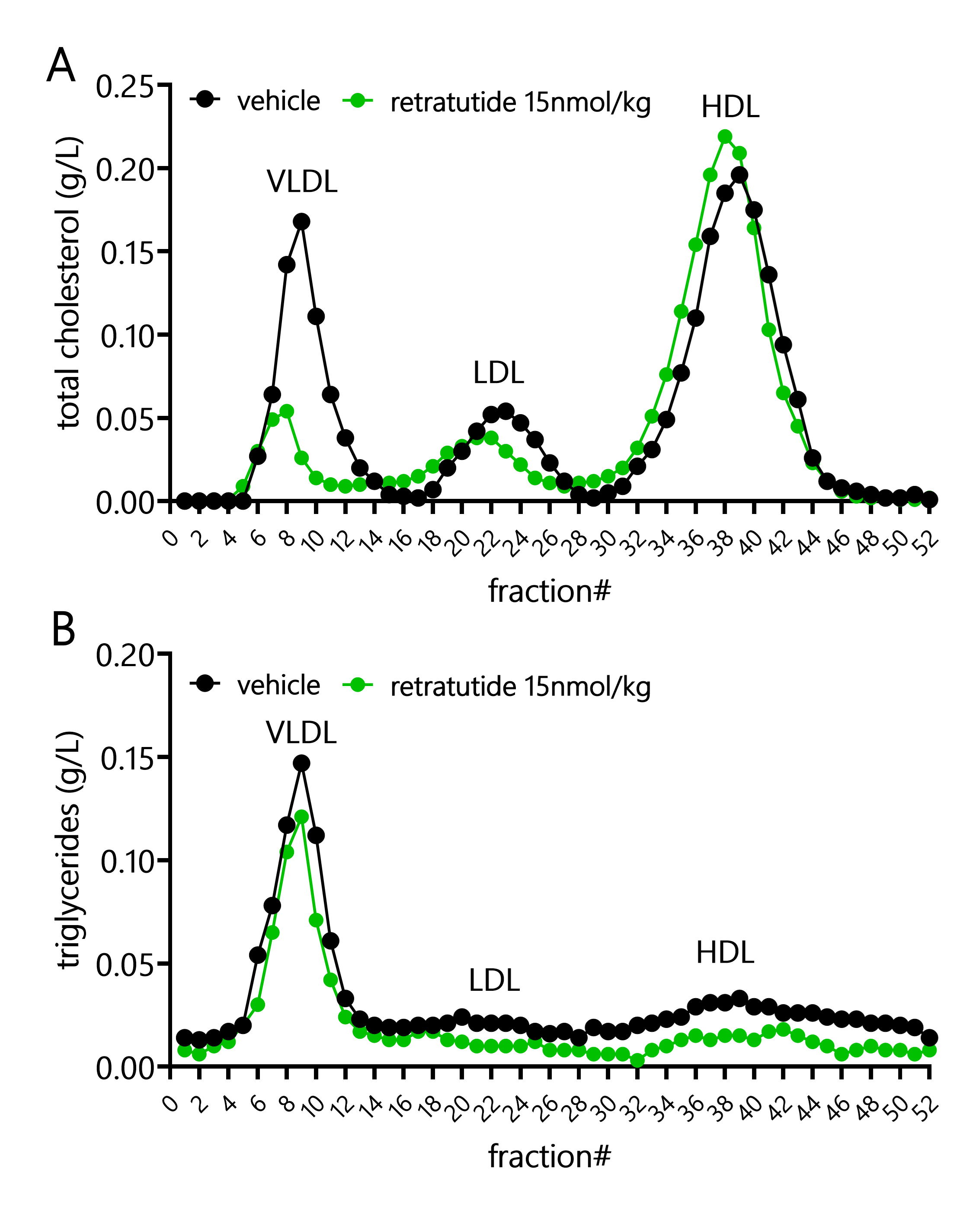

Supplement: Supplementary file 1 — Figure S1: Retatrutide improves dyslipidemia in free choice diet fed hamsters. (A) total cholesterol and (B) triglycerides lipoprotein profiles measured by fast protein liquid chromatography at the end of the 5‐week treatment with vehicle or retatrutide in free choice diet fed hamsters. [file OBY-34-43-s001.tif]
