# Supplementary material for: Retatrutide Shows Multiple Metabolic Benefits in Diet‐Induced Obese MASH Mouse and Hamster Models
Source: Obesity (Silver Spring). 2026 Feb 25;34(Suppl 1):43–53. doi: 10.1002/oby.70155 (PMC13250738; doi:10.1002/oby.70155)
Supplement: Supplementary file 2 — Table S1: Mouse qPCR primer sequences. [file OBY-34-43-s003.docx]

**Supplemental Table S1.** Mouse qPCR primer sequences

| Gene | GenBank ID | Forward Sequence (5’-3’) | Reverse Sequence (5’-3’) |
| --- | --- | --- | --- |
| Ppia | NM_008907 | CAAATGCTGGACCAAACACAA | GCCATCCAGCCATTCAGTCT |
| Cyc1 | NM_025567 | AACGCATGGGTCTCAAGATGT | ATGCCGCTTCATGGCATAG |
| Srebf1 | NM_011480 | AGTCATCTCTGTGCACCAGCAT | ACAAACAGGGTTCCCAGTCTACTC |
| Scd1 | NM_009127 | ACTCTGGGAGAGTGCTGACAAAA | TCACCTCAGAACTGCCCTTGA |
| Acaca | NM_133360 | ATTGACCCAGACTGGCTTGAA | GTGTGAAGGCTGCTTTGTGAAC |
| Acly | NM_001199296 | CAAAAACCCCCTCCTCAACA | TCCAGGCCCAGCCTATACTG |
| Cpt1a | NM_013495 | AGCAACTCCGTTTGGTATTC | ACAGGATGGTAACAGGTAGG |
| Il6 | NM_031168 | TACTCGGCAAACCTAGTGCGT | ATTTTCTGACCACAGTGAGGAATG |
| Il1b | NM_008361 | TCGCTCAGGGTCACAAGAAA | TCAGAGGCAAGGAGGAAAACAC |
| Tnf | NM_013693 | GGCACTCCCCCAAAAGATG | GCCACAAGCAGGAATGAGAAG |
| Ccl2 | NM_011333 | CACCAGCAAGATGATCCCAAT | TCTTTGGGACACCTGCTGC |
| Adgre1 | NM_010130 | CATCATTGCGGGATTCCTACA | GCATCACTGCCTCCACTAGCA |
| Acta2 | NM_007392 | GCACCCAGCACCATGAAGA | GAGCCACCGATCCAGACAGA |
| Timp1 | NM_011593 | TGCACAGTGTTTCCCTGTTTATCTA | CCTGATCCGTCCACAAACAGT |
| Col1a1 | NM_007742 | GCAGACTGGCAACCTCAAGAA | TGCCTTCGCCTCTGAGCT |
| Tgfb1 | NM_011577 | CATGCCAACTTCTGTCTGGGA | GCAAGGACCTTGCTGTACTGTGT |
