# Supplementary material for: Retatrutide Shows Multiple Metabolic Benefits in Diet‐Induced Obese MASH Mouse and Hamster Models
Source: Obesity (Silver Spring). 2026 Feb 25;34(Suppl 1):43–53. doi: 10.1002/oby.70155 (PMC13250738; doi:10.1002/oby.70155)
Supplement: Supplementary file 3 — Table S2: Hamster qPCR primer sequences [file OBY-34-43-s002.docx]

**Supplemental Table S2.** Hamster qPCR primer sequences

| Gene | GenBank ID | Forward Sequence (5’-3’) | Reverse Sequence (5’-3’) |
| --- | --- | --- | --- |
| Nono | XM_005081204 | TTGGCCAAGCTGCTACAATG | AGCTGGACGGTTGAATGCA |
| Eef1a1 | XM_005073705 | CAAAAATGACCCACCAATGGA | TTTGGCCTGGATGGTTCAG |
| Cyp7a1 | XM_005066730 | AGCAACTGACTGTGCCTAGGAAA | GGAACTCAGGCAGTGAGAACAGA |
| Fas | XM_013112078 | GGCAACTCCTGGTATGTTCACTTC | CCTTCTGGCCATTTTACCTTTTCT |
| Cpt1a | XM_005063328 | TAGGCATGAACGCAGAGCAC | GGCCATGACATACTCCCACAG |
| Scarb1 | XM_005080604 | AAGGCATCCCCATCTATCGC | CATTGGGTGGGTAGACGGAC |
| Ldlr | XM_005078536 | CGCCTCTGTGACCACTGTGT | GCCGCTGAGCTTGGTCC |
| Col1a1 | XM_005075850 | CAGCCTACTTCCCCACCTAGC | AGGCTCCTTCAAAAGTCCAAGA |
| Ccl2 | XM_005076967 | TGCTAACTTGACGCAAGCTCC | AAGTTCTTGAGTCTGCGGTGG |
| Il1b | XM_005068610 | GAAGTCAAAACCAAGGTGGAGTTT | TCTGCTTGAGAGGTGCTGATGT |
| Acta2 | XM_005063676 | CTGGTGGTCAAGCAAGGATGT | GGCTTCCCCTGTTTCTGTGAT |
| Il6 | XM_005087110 | CCATGAGGTCTACTCGGCAAA | GACCACAGTGAATGTCCACAGATC |
| Timp1 | XM_005085713 | TGTTCAACCATCCCTTGCAA | GAGCCCATGAGGATCTGATCTG |
| Casp3 | NM_001281582 | AGGTCCAAGTCACCACATGAGA | CACCACTTTGGGAAAGCCCT |
| Acaca | XM_013118312 | GAAGTCTCTTCTGTGGACGAGGA | GGAATAGGGATGGGTAACTTTGC |
| Col3a1 | XM_005084479 | TTCAAGATCAACACTGAGGAGATA | CTAATGAGGCTTTCTATTTGTCCAT |
